# Zymotic Diseases

**Published:** 1872-05

**Authors:** 


### ZYMOTIC DISEASES

Are such as result from 1st, Bad food; 2d, Bad air; 3d, Filth; 4th, Contagion. As the word "Zymotic," introduced within a few years, is so commonly employed in medical reports, especially those connected with cities and large towns, persons of cultivation and intelligence would do well to obtain a comprehensive and accurate knowledge of the meaning of the word, and fix it in the memory; it is from a Greek word which means to ferment; there can be no fermentation where there are not three conditions:—

1st. There must be moisture.

2d. There must be heat.

3d. There must be some growth which can decay.

If either one of these be absent, there can be no fermentation, which word may be considered in this connection equivalent to rotting, putrefying, decaying, decomposition; all these terms bring to our mind the idea of filth and dirt. The fundamental meaning, then, of the word zymotic, is filth; and all diseases arising from filth are diseases of zymotic origin. But different kinds of filth, which is really absence of cleanliness, cause different kinds of diseases. If a man has a house near a pond, and there are a great many leaves in it and about it, and logs of wood, and there is a hot sun, these are the three conditions; and in a short time the persons living near that pond will be attacked with fever, dysentery, diarrhœa, running through the whole neighborhood.

If a man has small-pox, ship fever, jail fever, the emanations from his body and his breath fill the air around him; any one coming within a few feet of him will take the disease, because of the filthiness of the atmosphere; in a measure you come in contact with him; hence these diseases are called contagious.

If you sleep with a man who has the itch, or sleep in a bed which he has just occupied, there is an idea of filthiness in the mind; there is a general feeling that people who have the itch are dirty people; hence itch is classed among zymotic diseases, and is also a contagious disease, because if you come in contact with such a person you will have the itch too, not directly from filth itself, but from that which is dependent on what we call filth, to wit: insects or growth of some kind; hence this kind of zymotic disease arises from parasites, and may be called parasitic disease, of which there are now supposed to be other kinds than that of common itch.

Zymotic diseases then are those which result from a want of cleanliness of person or habitation, and are avoidable. But the deaths from zymotic diseases, avoidable diseases, diseases which arise from filth, occurring in Philadelphia, for the week ending February 10th, 1872, were two hundred and ten out of five hundred and ten; that is, two fifths of all the deaths in so pleasant a city as Philadelphia, result from the want of cleanliness. If these statements have the effect on men of intelligence and influence to inquire more particularly into the connection between filth and death, it may lead to results ultimately which will benefit and bless all of human kind.

